# Concepts and methods for the dosimetry of radioembolisation of the liver with Y-90-loaded microspheres

**DOI:** 10.3389/fnume.2022.998793

**Published:** 2022-09-15

**Authors:** Arnaud Dieudonné, Manuel Sanchez-Garcia, Aurélie Bando-Delaunay, Rachida Lebtahi

**Affiliations:** ^1^ Department of Nuclear Medicine, Beaujon Hospital, APHP, Nord, University of Paris Cité, Clichy, France; ^2^ Department of Nuclear Medicine, Henri Becquerel Center, Rouen, France; ^3^ Servicio de Radiofisica y Proteccion Radiologica, Complexo Hospitalario Universitario de Santiago de Compostela, Santiago de Compostela, Spain

**Keywords:** radioembolisation, dosimetry, radiobiology, yttrium 90, microspheres

## Abstract

This article aims at presenting in a didactic way, dosimetry concepts and methods that are relevant for radio-embolization of the liver with ^90^Y-microspheres. The application of the medical internal radiation dose formalism to radio-embolization is introduced. This formalism enables a simplified dosimetry, where the absorbed dose in a given tissue depends on only its mass and initial activity. This is applied in the single-compartment method, partition model, for the liver, tumour and lung dosimetry, and multi-compartment method, allowing identification of multiple tumours. Voxel-based dosimetry approaches are also discussed. This allows taking into account the non-uniform uptake within a compartment, which translates into a non-uniform dose distribution, represented as a dose–volume histogram. For this purpose, dose–kernel convolution allows propagating the energy deposition around voxel-sources in a computationally efficient manner. Alternatively, local-energy deposition is preferable when the spatial resolution is comparable or larger than the beta-particle path. Statistical tools may be relevant in establishing dose–effect relationships in a given population. These include tools such as the logistic regression or receiver operator characteristic analysis. Examples are given for illustration purpose. Moreover, tumour control probability modelling can be assessed through the linear-quadratic model of Lea and Catcheside and its counterpart, the normal-tissue complication probability model of Lyman, which is suitable to the parallel structure of the liver. The selectivity of microsphere administration allows tissue sparing, which can be considered with the concept of equivalent uniform dose, for which examples are also given. The implication of microscopic deposition of microspheres is also illustrated through a liver toxicity model, even though it is not clinically validated. Finally, we propose a reflection around the concept of therapeutic index (TI), which could help tailor treatment planning by determining the treatment safety through the evaluation of TI based on treatment-specific parameters.

## Introduction

Treatment planning of selective internal radiation therapy (SIRT) of the liver with ^90^Y-microspheres is a key step for the determination of administered activity aiming to balance the risk and benefit of treatment. For both devices currently available for ^90^Y-SIRT, glass and resin microspheres, specific recommendations that rely on dosimetry for treatment planning were recently proposed by panels of experts (1, 2). Indeed, despite the fact that both products use the same radionuclide, the different average activities per sphere, 2,500 Bq/sphere for glass and 50 Bq/sphere for resin microsphere, lead to different recommended efficacy and toxicity thresholds. For example, the treatment of unilobar hepatocellular carcinoma (HCC) would require targeting at least 205 Gy to the tumour while limiting the dose to the whole normal liver to 75 Gy if glass microspheres are used (1) and at least 100–120 Gy to the tumour and a maximum of 40 Gy to the whole normal liver for resin microspheres (2).

For both microsphere types, this pretherapeutic dosimetry is based on the intra-arterial injection of ^99m^Tc-macro-aggregated albumin (^99m^Tc-MAA) into the same catheter position that will later be used for the therapeutic injection of ^90^Y-microspheres. Several methods are available to compute the pre-therapeutic absorbed dose for activity planning. Needless to say, the so-called body surface area method, which was part of resin microspheres treatment planning, shall not be confused with the following dosimetry systems as it is only a tool for activity planning.

A common dosimetry method is the partition model, proposing a dosimetry formalism from quantification of lung shunt fraction (LSF) and tumour-to-normal-liver (T/N) ratio of ^99m^Tc-MAA hepatic and tumour deposition. With these inputs, the method allows calculating the absorbed dose to lung, normal liver, and tumour.

The spread of 3D quantification in nuclear medicine routine practice has enabled multi-compartmental dosimetry, providing absorbed dose values in as many compartments as needed (liver, non-tumoral liver, treated liver, tumours) and greater degree of personalisation (3). A further personalisation level is achieved with voxel-level dosimetry, i.e., 3D dosimetry, where the absorbed dose can be represented as dose-volume histograms or isodose curves.

The availability of adequate methodology and cohort selection has enabled several retrospective analyses where a dose–effect relationship with tumour response and/or survival (4–8) has been established. Establishing a dose–effect relationship has two final objectives: (1) early prediction of an outcome and (2) better tailoring of treatment planning. Receiver operator characteristic (ROC) analysis and logistic regression are statistical tools that help describe this dose–effect relationship, but radiobiological modelling can also be used from a mechanistic point-of-view for this purpose. This is possible with the tumour control probability (TCP) based on the linear-quadratic model of Lea and Catcheside (9) and its counterpart the normal-TCP (NTCP) model of Lyman. The selectivity of microsphere administration allows tissue sparing, which can be considered with the concept of equivalent uniform dose (EUD). The implication of microscopic deposition of microspheres is also illustrated through liver toxicity model, even though it is not clinically validated. The concept of therapeutic index (TI) could also help to better tailor treatment planning by determining the treatment safety through the evaluation of TI based on treatment-specific parameters.

Recently, the European Association of Nuclear Medicine committees of therapy, oncology, and dosimetry have published recommendations for patient care and pre- and post-therapeutic dosimetry implementation of SIRT of the liver with ^90^Y-microspheres (10, 11). We do not aim to substitute these recommendations but to present in a didactic way, dosimetry concepts and methods that are relevant for radio-embolization of the liver with 90Y-microspheres.

## Region-based dosimetry

### Application of the MIRD formalism

In region-based dosimetry, regions of interest are defined, typically the tumour, liver, and lung. The MIRD methodology allows computation of dose on any target region *r_T_*, *D*(*r_T_*), according to Equation 1 where $\tilde{A}(r_{S})$ is the time-integrated activity in the source region $r_{S}$. $\tilde{A}(r_{S})$ is calculated from the integration of $A(r_{S},t)$, which describes activity in $r_{S}$ over time (12):

(1) $$D(r_{T})=\underset{r_{S}}{\sum }\tilde{A}(r_{S})\times S(r_{T}\leftarrow r_{S})$$

In SIRT, $\tilde{A}(r_{S})\;$ can be simply calculated as $\tilde{A}(r_{S})=A(r_{S},0)/\lambda_{Y90}$ with $A(r_{S},0)$ being the activity at *t* = 0 and $\lambda_{Y90}$ being the decay constant for ^90^Y. This is possible due to absence of biological elimination.

Introducing $\varphi (r_{T}\leftarrow r_{S})$ as the fraction of emitted energy in $r_{S}$ that is absorbed in $r_{T}$, it can be shown that

(2) $$S(r_{T}\leftarrow r_{S})=\;\Delta_{Y90}^{\beta }\times \varphi (r_{T}\leftarrow r_{S})$$

where $\Delta_{Y90}^{\beta }$ is the average energy released per decay. Finally, Equation 1 becomes

(3) $$D(r_{T})=\;\frac{\Delta_{Y90}^{\beta }}{\lambda_{Y90}\times M(r_{T})}\underset{r_{S}}{\sum }A(r_{S},0)\times \varphi (r_{T}\leftarrow r_{S})$$

with $\Delta_{Y90}^{\beta }=933\;\mathrm{keV}$ and $\lambda_{Y90}=3.00\times 10^{-6}\;s^{-1}$, $M(r_{T})$ being the mass of $r_{T}$. Note that $\Delta_{Y90}^{\beta }/\lambda_{Y90}$ is a radionuclide-specific constant that, for SIRT, gives the average energy released per Bq of injected activity, numerically:

(4) $$\frac{\Delta_{Y90}^{\beta }}{\lambda_{Y90}}=311\;keV.s=49.7\times 10^{-9}\;J.Bq^{-1}$$

For simplicity, we will use the rounded value 50 for the following equations.

Therefore, $D(r_{T})$ in Gy can be expressed as

(5) $$D(r_{T})[Gy]=\frac{50\;}{M(r_{T})[kg]}\underset{r_{S}}{\sum }A(r_{S},0)\;[GBq]\times \varphi (r_{T}\leftarrow r_{S})$$

With the hypothesis of non-penetrating beta-particles and negligible bremsstrahlung radiation, all energy released in a region is absorbed in the same region, i.e., $\varphi (r_{T}\leftarrow r_{S})=\varphi (r_{S})=1$, Equation 5 becomes

(6) $$D(r_{S})[Gy]=\frac{50}{M(r_{T})[kg]}A(r_{S},0)[GBq]$$

This hypothesis has been extensively investigated for spherical volumes, with self-absorbed fraction in spheres of varying sizes readily available in the literature (13, 14).

With some degree of approximation, count losses due to partial volume effect, which are present in positron emission imaging (PET) and single photon computed emission imaging (SPECT), mimic the transport of energy by beta-particles out of voxels, and consequently, a local energy deposition approximation (*φ* = 1) may remain valid for spatial resolutions >5 mm (15, 16).

**Figure 1:**
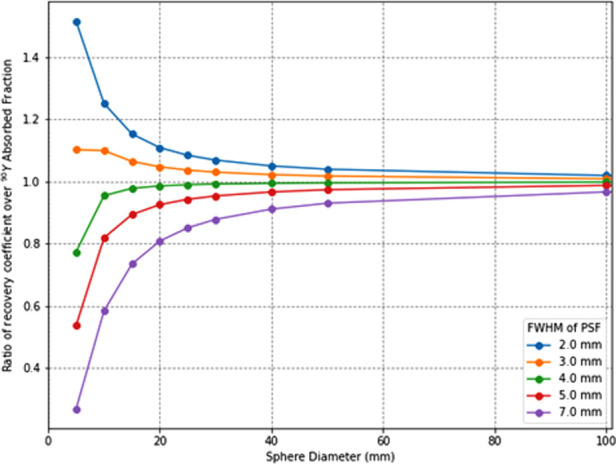
Ratios of recovery coefficients over ^90^Y soft tissues (1.04 g/cm^3^) absorbed fractions for spatial resolutions (point-spread-function full-width-at-half-maximum) from 2 to 7 mm.

To verify how accurately the system point spread function (PSF) mimics the energy transport of particles, one can calculate the ratio of the recovery coefficient (RC) over the absorbed fraction $\mathrm{RC}(r_{S})/\varphi (r_{S})$. RC is the ratio of the signal intensity degraded by the PSF over the ideal intensity. Its value is between 0 and 1 as for the absorbed fraction. $\mathrm{RC}(r_{S})/\varphi (r_{S})=1$ means that the mimicking is perfect whereas, for a ratio < 1, the PSF degradation is “stronger” than the particle transport and that there is a residual partial volume effect. Inversely, a ratio >1 means that the particle transport is not fully mimicked by the PSF. To illustrate this, the ratio for spheres of various diameters and for various PSFs full width at half maximum (FWHM) has been calculated. The computed values are compiled in Table 1 and plotted in Figure 1. This shows that, for the smallest spheres (diameter <20 mm), the ratio diverges from 1 and that for the greatest it converges to 1 (diameter >50 mm), even for larger FWHM values.

**Table 1.** Ratio of recovery coefficients over ^90^Y absorbed fraction in soft tissues (1.04 g/cm^3^). The closer is the ratio to one, the better is the accuracy by considering a local energy deposition approximation (*ϕ* = 1).

Sphere diameter (mm)	FWHM of PSF				
	2 mm	3 mm	4 mm	5 mm	7 mm
5	1.52	1.10	0.77	0.54	0.27
10	1.25	1.10	0.95	0.82	0.58
15	1.15	1.06	0.98	0.89	0.73
20	1.11	1.05	0.99	0.93	0.81
25	1.08	1.04	0.99	0.94	0.85
30	1.07	1.03	0.99	0.95	0.88
40	1.05	1.02	0.99	0.97	0.91
50	1.04	1.02	1.00	0.97	0.93
100	1.02	1.01	1.00	0.99	0.97
200	1.01	1.00	1.00	0.99	0.98

For each sphere of diameter *d*, RC(d,FWHM) was calculated by convoluting an image I(d) containing the value “0” outside the sphere SPH of diameter *d* and “1” inside the sphere with Gaussian functions g centred on 0 and with a FWHM ranging from 2.0 to 7.0 mm. Then, the resulting image I(d,FWHM)=I(d)⊗g(FWHM) is used to calculate RC(*d*, FWHM) = Σ_SPH_ *I*(*d*, FWHM) / Σ_SPH_ *I*(*d*). The absorbed fraction φ(d) was calculated by convoluting I(d) to the dose kernel of ^90^Y in water scaled to soft-tissue density $k_{Y90}^{w}$. $k_{Y90}^{w}$ was previously calculated with Monte Carlo simulations (17). The resulting image $D(d)=I(d)⊗k_{Y90}^{w}$ is the absorbed dose distribution for a sphere source of diameter *d*; thus, the absorbed fraction is given by $\varphi (d)=\sum_{SPH}D(d)/\left(\sum_{SPH}I(d)\times \Delta_{Y90}\right)$.

### Partition model

The partition model (18) was proposed to consider the liver-to-lung shunt and different specific activities of the tumour and the remaining targeted hepatic parenchyma. It implies the measurement of LSF and T/N ratio, leading to a three-compartment dosimetry model where all injected activities are assumed to end up in three regions, namely, the liver, tumour, and lung. As initially proposed, the LSF and T/N ratio *r* are computed from 2D measurements on planar hepatopulmonary scintigraphy after ^99m^Tc-MAA intra-arterial injection to simulate the distribution of ^90^Y-microspheres.

For LSF, the total number of counts in the lung and liver is used:

(7) $$LSF=\frac{A_{L}}{A}=\frac{total\;number\;of\;counts\;in\;lung}{\begin{array}{c} total\;number\;of\;counts\;in\;lung\\ +total\;number\;of\;counts\;in\;liver \end{array}\;}$$

where $A_{L}$ is the activity in the lung at time of administration and *A* is the administered activity.

The T/N is computed from the average uptake in the tumour and non-tumoral liver:

(8) $$r=\frac{\frac{A_{T}}{M_{T}}}{\frac{A_{N}}{M_{N}}}=\frac{average\;number\;of\;counts\;in\;tumor}{average\;number\;of\;counts\;in\;liver}$$

where $A_{T}$ and $A_{N}$ are the activities in the tumour and normal liver, respectively, at time of administration and $M_{T}$ and $M_{N}$ are the corresponding masses. Consequently, the activity in the tumour can be calculated from the administered activity *A* with

(9) $$A_{T}=\frac{M_{T}\times r}{M_{N}+M_{T}\times r}\times (1-LSF)\times A$$

and the activity in normal liver $A_{N}$ can be deduced as

(10) $$A_{N}=(1-LSF)\times A-A_{T}$$

Trivially, activity in the lung is obtained as

(11) $$A_{L}=LSF\times A$$

Then, the absorbed $D_{r}$ to tumour, normal liver, and lungs can be calculated from the corresponding activity $A_{r}$ by

(12) $$D_{r}[Gy]=\frac{50\times A_{r}\;[GBq]}{M[kg]}$$

It should be noted that, with this approach, only the absorbed dose for one tumour is calculated or averaged in multiple tumours.

### Multi-compartmental model

The widespread availability of 3D quantification through SPECT devices and iterative reconstructions with CT-based attenuation correction and scatter compensation has enabled higher accuracy in the quantification of relative activity between organs (19), with the ability to prevent organ overlap compared to planar imaging. It is possible to apply the direct measure of the relative activity, for an arbitrary number (N) of compartments, segmented in the emission images, to the MIRD formalism. In the obtained multi-compartmental approach, all injected activities are assumed to end up in N compartments. For each compartment *c*, the fraction of injected activity (FIA, $a_{c}$) and mass $\left(M_{c}\right)$ are measured and used as input in the MIRD formalism to compute the pre-therapeutic absorbed dose per unit of administered activity or absorbed dose coefficient $\left(d_{c}\right)$.

(13) $$d_{c}[Gy.GBq^{-1}]=\frac{a_{c}\times 50}{M_{c}\;[kg]}$$

The main difference between the three-compartment (partition) and multi-compartment models is the quantification step. On one side, quantification relies on 2D or 3D measurements of LSF and T/N ratio, for a single tumour or averaged over multiple tumours. On the other side, it is based on the 3D measurement of the FIA on any given compartment, such as the tumour(s), non-tumoral liver, and targeted liver. Table 2 summarises the differences between both methods.

**Table 2:** Comparison of the formalisms derived from the MIRD formula.

	MIRD single compartment	Partition model	MIRD multi-compartment
Compartments	Treated liver	Lung *L*, normal liver *N* and tumour *T*	Lung, normal liver, treated liver, tumour(s), etc.
Calculation method	n/a	$A_{L}=A\times LSF$	$a=\frac{N}{N_{T}+N_{N}+N_{L}}$
		$A_{T}=\frac{r\;\times A_{N}\times M_{T}}{M_{N}}$	$a_{T}+a_{N}+\;a_{L}=1$
		$A_{N}=\frac{A_{T}\times M_{N}}{r\times M_{T}}$	
Pre-therapeutic absorbed dose calculation	In any given compartment, the absorbed *D* is derived from the initial activity A within the compartment and its mass M.		The absorbed coefficient *d* is derived from the fraction of injected activity *a* and its mass *M*.
post-therapeutic absorbed dose calculation	$D[\mathrm{Gy}]=\frac{A[\mathrm{GBq}]\times 50}{M[\mathrm{kg}]}$		$d[\mathrm{Gy}.\mathrm{GB}q^{-1}]=\frac{a\times 50}{M[\mathrm{kg}]}$
			The absorbed dose *D* is derived from the activity concentration *c*_0_.
			$D[\mathrm{Gy}]=c_{0}\;[\mathrm{Bq}.ml^{-1}]\times \frac{50\times 10^{-6}}{\rho [g.cm^{\left(-3\right)}]}$

More recently, ^90^Y-PET imaging has been applied to SIRT, allowing direct absolute quantification of the post-treatment activity concentration at the voxel level (in Bq.mL^−1^). Thus, the average absorbed dose can be calculated by implementing the MIRD formula to PET quantification:

(14) $$D[Gy]=c_{0}\;[Bq.cm^{-3}]\times \frac{50\times 10^{-6}}{\rho [g.cm^{-3}]}$$

With $c_{0}$ being the concentration in any given tissue at the time of administration.

## Voxel-based dosimetry

**Figure 2:**
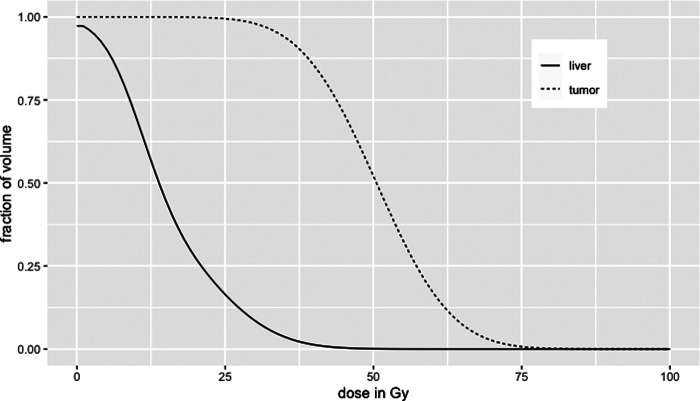
Typical tumour and liver cumulative dose–volume histograms in ^90^Y radioembolisation.

Voxel-based dosimetry aims at providing a 3D distribution of the absorbed dose, reflecting absorbed dose inhomogeneity within compartments accessible on the macroscopic scale allowed by the scanner spatial resolution. 3D dose distributions are usually presented as isodose curves superimposed on anatomical imaging or summarised using a dose-volume histogram (DVH). Typical DVH shapes are shown in Figure 2. A DVH represents the volume-to-absorbed dose distribution within a given compartment according to several dose intervals or bins. The DVH is usually expressed in its cumulative form, providing information on the fraction of the considered compartment volume that receives at least a given amount of absorbed dose. Then, characteristic values can be extracted such as D_X_ as the least absorbed dose irradiating *X* % as the volume of the considered compartment or V_YGy,_ as the volume receiving at least an absorbed dose of *Y* Gy. The DVH can be also qualitatively interpreted by analysing its shape. A rectangular DVH indicates a homogeneous irradiation.

### Model-based energy deposition

Approaches based on the convolution of a dose kernel (either using point or voxel sources) were proposed for taking into account radionuclide non-uniform distribution in a homogenous medium (20–23). The dose-point kernel plots the energy deposited around a source according to the distance to the source and can be calculated analytically or using Monte Carlo simulations (24–26). Tissue density heterogeneities can be considered by scaling dose-point kernel linearly according to the radiological distance between sources and targets, i.e., the distance travelled by particles in water for the same energy deposition. The superposition of dose-point kernels can be accelerated using the collapsed cone approximation, where a finite number of directions around the source is considered for energy deposition, thus reducing the complexity of the calculation (17).

The dose-point kernel convolution considers the sources and targets as points at the centre of each voxel, though corrections are available to model the finite dimension of voxels. Moreover, dose-voxel kernel convolution, also known as voxel S-values, directly models sources and targets as voxels (27), losing the ability of performing radiological distance scaling. Dose-voxel kernels are calculated from integration of dose-point kernels or using direct Monte Carlo simulations (28–30). Once a fine resolution dose-voxel kernel is available, resampling approaches have been developed to adapt it to any voxel dimensions (31, 32).

### Monte Carlo simulation

Monte Carlo simulation is the method of reference for calculating energy deposition around a source, be it extended or point-like, and remains the gold standard for 3D dosimetry (33–36). Software packages are currently available, which easily translate patient-specific voxel information (such as activity concentration and tissue density at the voxel level) in Monte Carlo codes (33, 37–39). It allows increasing the accuracy on the absorbed dose deposition in the presence of tissue heterogeneities, thanks to a comprehensive modelling of particle interaction with the matter, while other approaches rely on a simplified modelling but with less computationally demanding calculations.

### Local energy deposition

Similarly to the region-based dosimetry, the hypothesis of non-penetrating particles can be done at the voxel level. The continuous slowing down approximation (CSDA) range of ^90^Y electrons, close to the mean range, is 3.85 mm in soft tissues (*ρ* = 1.04 g.cm^−3^) and 16 mm in the lung (*ρ* = 0.25 g.cm^−3^) (40). This approximation is used to calculate the electron range by assuming the continuous loss of energy by electrons from their initial kinetic E_0_ energy to rest. The CSDA range is calculated by integration the inverse of the total stopping power according to the energy from E_0_ to 0. The negligible bremsstrahlung emission (< 1% of E_0_) indicates little energy is transported farther than the electron range.

**Figure 3:**
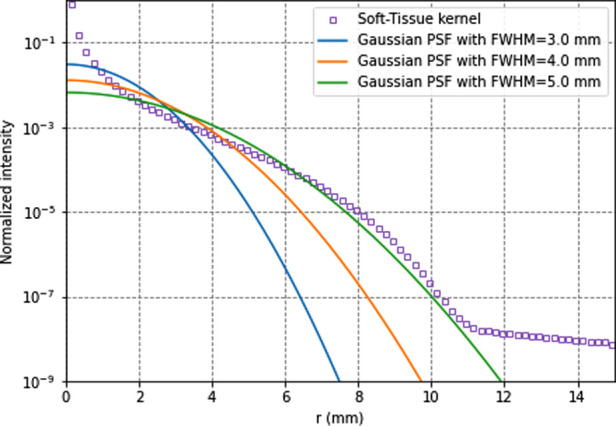
^90^y soft-tissues dose-point kernel and point-spread functions for full-width-at-half-maximum of 3.5, 4.5, and 5.5 mm. All data are normalised for comparison purpose.

Figure 3 compares Monte Carlo-generated soft-tissue dose point kernel (17) (DPK) and PSFs for several spatial resolutions. It can be seen that, for the soft tissue, the DPK curve and 4.5-mm PSF are very similar for distances >2 mm. Hence, any system PSF with FWHM >4.5 mm mimics the energy transport. The absorbed dose in voxels can thus be more accurately calculated with the local-energy deposition method than with convolution methods. Moreover, there is still a remaining partial volume effect that should be accounted for, for better accuracy.

Therefore, the absorbed dose in voxels can be calculated using Equation 12.

In a soft-tissue medium and since all voxels resulting from a SPECT or PET reconstruction have the same volume $V_{v}$, the voxel-based absorbed calculation is the result of this calculation for all voxels:

(15) $$D_{v}[Gy]=\frac{A_{v}[Bq]}{V_{v}[cm^{3}]}\times 48\times 10^{-9}$$

## Dose-effect relationship

Here we present and discuss methods for establishing dose–effect relationship starting from the same dataset.

**Figure 4:**
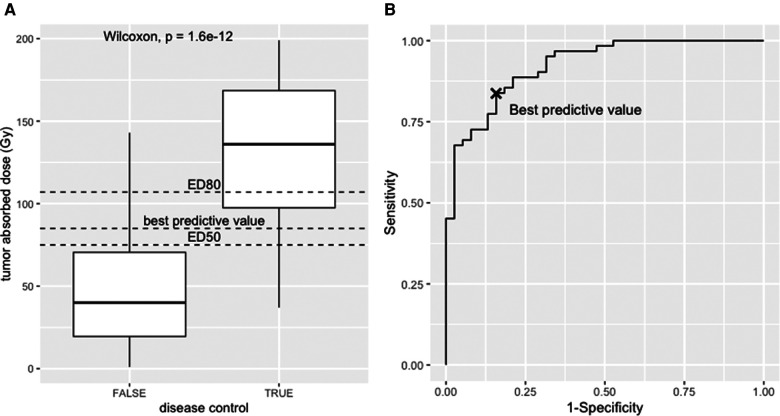
(**A**) Box-plot, responders vs. non-responders towards the tumour absorbed. (**B**) Receiver operator curve analysis of the simulated dataset.

The dataset was generated as follows: 100 lesions with absorbed dose ranging from 1 to 199 Gy (one every 2 Gy) were classified as responders and non-responders (Figure 4) with a binomial random variate generated with the software tool R (41), following a sigmoidal probability distribution:

(16) $$Probability\;of\;response\;(D)=e^{-8\times e^{-0.03\times D\times (1-0.000033\times D)}}$$

The dataset and the code to generate it are provided as Supplementary data. The median absorbed dose was 136 Gy for responders and 40 Gy for non-responders. The statistical significance was tested using the nonparametric Wilcoxon test (*p* < 0.001). See boxplot on Figure 4A.

### Statistical tools

The receiver operator characteristic (ROC) analysis evaluates the performance of the tumour-absorbed dose as a response predictor. The ROC curve plots the true positives rate (sensitivity) towards false positive rate (1—specificity) using the ROCR package (42). The ROC curve of the provided dataset is shown in Figure 4B, with an area under the curve (AUC) of 0.92 (95% CI, 0.87–0.97). This means that the model has 92% chance to separate responders from non-responders. From this example, the best predictive value determined using the Youden index is 85 Gy (sensitivity = 0.84, specificity = 0.84), i.e., conventionally the best discriminant between responders and non-responders, corresponding to the maximum sum of sensitivity and specificity. Interestingly, this value has a probability of response of 61% according to the logit model.

For a categorical variable, such as the occurrence of response or complications, the logistic regression mathematically models the probability of the effect *P* towards any variable, following this equation:

(17) $$P(D)=\frac{e^{-aD+b}}{1+e^{-aD+b}}$$

With a and b being fit parameters.

**Figure 5:**
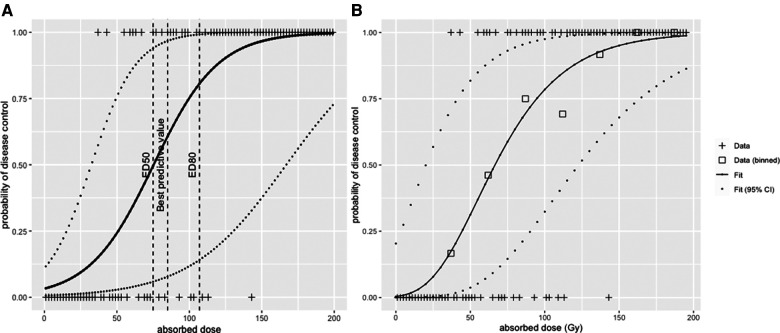
(**A**) logistic regression and (**B**) radiobiological linear quadratic model fitted to the simulated dataset.

The result of a logistic regression for the dataset is shown in Figure 5A. From this modelling, the median efficacy dose, i.e., the dose that leads to a 50% probability of response, was ED_50_ = 75 Gy (95% confidence interval—CI, 31–167 Gy), and the dose that leads to a 80% efficacy dose ED_80_ = 107 Gy (95% CI, 55–213 Gy).

This illustrates that according to the tool that is used, the result will have a different interpretation. The ROC curve tests the performance of the absorbed dose as a classifier (AUC = 1, perfect classifier; AUC = 0.5, random classifier), the logistic regression model instead deals with the dose–response function from a mechanistic description of the response probability as a function of the absorbed dose.

### Radiobiological tools

The linear-quadratic (LQ) model has been extensively used to model the dose–survival relationship in external beam radiation therapy. While it is supported by radiobiological concepts, it can be used as a pure mechanistical model (43). In the following sections, we present the use of LQ models for the determination of the tumour control probability (TCP) and the normal tissue complication probability (NTCP) as a consequence of cell killing caused by a given amount of radiation absorbed dose in tissues.

#### Tumour control probability

According to the linear-quadratic (LQ) model, the probability *P* of killing a tumour with a given dose D is a function of a set of radiobiological parameters with

(18) $$P(D)=e^{-N_{0}e^{-\alpha D-G\beta D^{2}}}$$

with $N_{o}$ being the number of clonogenic cells, α and β are the linear and quadratic cell killing constants, respectively, and *G* is the Lea–Catcheside repair factor (9). In SIRT, because of the absence of activity re-localisation, it reduces to λ/(λ+μ) (44), where μ is the DNA repair constant and λ is the decay constant of ^90^Y, i.e. $\lambda =3\times 10^{-6}\;s^{-1}=0.0108\;h^{-1}$.

Values for α/β and μ can be found in the literature for various tissue types and are not specific to ^90^Y beta particles. Typical values used are α/β=10Gy and $\mu =0.46\;h^{-1}$ for tumours (45). However, these values remain controversial; for example, a value α/β=15Gy was also reported for HCC (46). The number of clonogenic cells $N_{0}$ is directly related to the volume of the tumour and suggests that tumour control depends on the dose and volume, which may complicate the modelling based on clinical data.

Figure 5 shows the fit of the LQ model to the data in Figure 4, with data binned into intervals of 25 Gy. Each bin was assigned a value that corresponds to the mean of all absorbed dose values within that bin. The fit was conducted with the Levenberg–Marquardt algorithm, giving that ED_50_ = 65 Gy (95% CI, 20–125 Gy) and ED_80_ = 105 Gy (95% CI, 50–175 Gy).

#### Equivalent uniform dose

To consider the biological effect of absorbed dose inhomogenities within the tumour or organs at risk, the EUD was proposed by Jones and Hoban (47, 48) to report the absorbed dose. The following model assumes a uniform distribution of clonogens within the tumour and that the organ-at-risk structure is parallel, which is the case for the liver.

For a tumour of N voxels the EUD is given by

(19) $$EUD=-\frac{1}{\alpha }ln\left(\frac{\sum_{i=1}^{N}e^{-\alpha D_{i}}}{N}\right)$$

Practically, EUD is calculated from the differential histogram of dose values over the volume considered. This does not take into account the hierarchical structure of an organ, which, in the case of liver, is not the problem itself, thanks to its parallel organ architecture (49, 50).

α is the tissue radiosensitivity, the highest the value, the highest the number of killed cell fraction for a fixed dose D. According to data published by Emami et al. (51), α should be 0.0065 Gy^−1^ in the liver (52), whereas, for HCC tumours, different values were reported. Strigari et al. (44) calculated an *α*-value of 0.001 Gy^−1^ for radioresistant tumours and 0.005 Gy^−1^ for less radioresistant tumours in nonresectable HCC treated with resin microspheres. The TCP was fitted with response data (complete and partial vs. stable or progressive). With the same approach, Chiesa et al. (4) have found an *α*-value of 0.004 Gy^−1^ for intermediate and advanced HCC treated with glass microspheres. D’Abadie et al. (53) found *α*-values from 0.034 to 0.038 Gy^−1^ in HCC treated with resin or glass microspheres. The analysis differs from the aforementioned studies in that it uses a tumour EUD of 40 Gy, a cutoff used in EBRT as predictor of longer survival to stratify Kaplan–Meier curves of overall survival and maintain the best agreement between the two types of microspheres.

**Figure 6:**
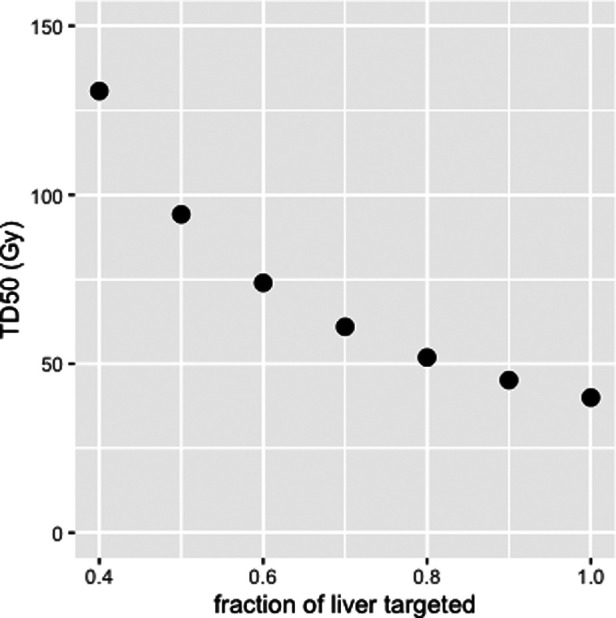
Median toxic dose (TD_50_), i.e., liver absorbed dose leading to a 50% probability of liver failure, as a function of liver fraction irradiated.

Figure 6 shows the TD_50_ of targeted liver (EUD = 40 Gy), corresponding to 50% probability of appearance of a liver failure in EBRT, for liver fraction *f* from 0.4 to 1.0 (51). This modelling shows that sparing the non-tumoral liver from radiation exposure allows for higher absorbed dose to the targeted liver without increasing the risk of liver failure. For example, for *f* = 0.7, TD_50_ (EUD = 40 Gy) of the targeted liver is 61 Gy, with liver absorbed dose = 43 Gy, whereas for *f* = 0.4, TD_50_ = 133 Gy, with liver absorbed dose = 53 Gy. One can see that, at least for *f* > 0.4, EUD is extremely close to the liver absorbed dose, which might explain why Chiesa et al. (54) refers to the non-tumoral whole liver absorbed dose (NTWLD) to established dose constraints to limit liver decompensation. NTWLD is a dosimetric parameter that is easily available through region-based dosimetry.

#### Normal tissue complication probability

Clinical data supports that the liver risk of complication or radiation-induced liver disease is a function of the irradiated fraction and absorbed dose (55). The normal tissue complication probability (NTCP) proposed by Lyman (56)

(20) $$NTCP(t)=\frac{1}{\sqrt{2\pi }}\int_{-\infty }^{t}e^{\frac{-s^{2}}{2}}ds$$

where $t=((D-TD_{50}(v))/(m\times TD_{50}(v)))$, with *D* the absorbed dose to the fraction of liver irradiated (v), $TD_{50}(v)=TD_{50}/v$ and *m* the slope of the response curve near TD_50_. An attempt to consolidate both Jones and Hoban EUD and Lyman NTCP models has also been proposed (55).

#### Microscale considerations

Microspheres travel within the blood vessels results in dose inhomogenities at the microscopic level according to the concentration of microspheres per volume unit. The heterogeneity of sphere distribution was employed to better understand the relationship between liver absorbed dose and toxicity for the different types of spheres (57, 58). Walrand et al. (52) have proposed a micro-scale model based on statistical arterial tree branching and geometric lobules where the microspheres are trapped. This model reconciliates differences in dose thresholds for hepatic toxicity between external beam radiation therapy, resin and glass microspheres using the concept of EUD of Jones and Hoban. According to their model, the TD_50_ to the targeted liver can be expressed as a function of *v*, the liver targeted fraction, and msA, the activity per sphere in kBq:

(21) $$TD_{50}=\frac{25.2+22.1\times (1-e^{-2.74\times msA})}{{\left(v-0.4\right)}^{0.584}}$$

As an example, TD_50_ for a whole liver treatment (v=1) would be 38 Gy for resin microspheres (50 Bq/spheres) and 64 Gy for glass microspheres (2,500 Bq/spheres). For a treatment with a targeted liver fraction of 70% (v=0.7), the targeted liver absorbed dose is 57 Gy for resin and 96 Gy for glass microspheres. It must be noted that this model does not take into account potential liver dysfunction, neither previous treatments (chemotherapy, chemoembolization, SIRT). Consequently, it should be employed with care in these situations.

## Therapeutic index for optimal treatment tailoring

TI establishes a quantitative relationship between the efficacy and toxicity of a drug for a given population. The larger the TI, the better the trade-off between treatment safety and efficacy. Conversely, a low TI requires caution to prevent potential toxicity concomitant with possible low treatment efficacy. TI is commonly calculated as the ratio of median toxic (TD_50_) over median efficacy doses (ED_50_):

(22) $$TI=\frac{TD_{50}}{ED_{50}}$$

For personalised treatments, such as radioembolisation, TI can help understand how the treatment parameters have an influence on treatment safety and optimize the therapeutic gain (59).

If we express the TI according to the tumour absorbed dose, Equation 22 would become

(23) $$TI=\frac{{TD}_{50}^{tumor}}{{ED}_{50}^{tumor}}=\frac{{TD}_{50}^{liver}/d_{N}\times d_{T}}{{ED}_{50}^{tumor}}=\frac{{TD}_{50}^{liver}}{{ED}_{50}^{tumor}}\times \frac{d_{T}}{d_{N}}=\frac{{TD}_{50}^{liver}}{{ED}_{50}^{tumor}}\times r\;$$

where *r* is the T/N ratio and $d_{N}$ and $d_{T}$ are the absorbed dose coefficients of normal liver and tumour respectively.

Thus, the benefit/risk balance can be quantified by knowing T/N, $ED_{50},$ and $TD_{50}$. For a high T/N ratio and a low liver fraction, the treatment will provide a wide TI, contrary to a low T/N with a high fraction of liver treated.

To illustrate this, one can calculate the TI for a lobar treatment (*Vf* = 0.7) of a HCC with resin microspheres with *r = 2*. Looking at the results of Hermann et al. (7), the value of ${\mathrm{ED}}_{50}^{\mathrm{tumor}}$ for disease control was 70 Gy for resin microspheres. According to Walrand et al. (52), treatment with *Vf* = 0.7 and resin microspheres (msA = 50 Bq/sphere) would have ${\mathrm{TD}}_{50}^{\mathrm{liver}}=70\;\mathrm{Gy}$. Hence, TI=70/70×2=2 for this treatment, indicating that the activity leading to a 50% probability of liver complication is twice as large as the activity leading to 50% probability of tumour control. For *Vf* = 1.0 $\left(TD_{50}=55\;\mathrm{Gy}\right)$ and *r = 1.3*, TI=55/70×1.3=1.02. For a selective treatment (*Vf* < 0.4), according to the aforementioned model, TD_50_ and thus TI end to infinity, which in this case indicates that segment ablation is safe.

A more complex model is the complication-free TCP P* (60), which is expressed as the product of TCP by the probability of no complication (1-NTCP)

(24) $$P^{*}=TCP\times (1-NTCP)$$

**Figure 7:**
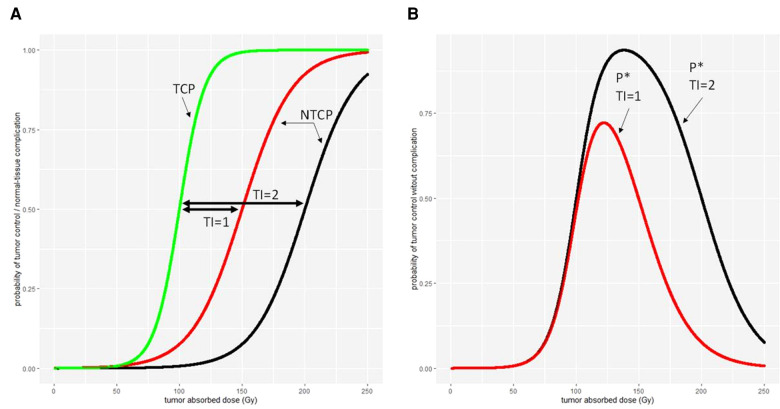
Different therapeutic indexes on (A) tumour control probability (TCP) and normal-tissue complication probability (NTCP) and (B) the tumour control without normal-tissue complication probability P*.

This model allows to compute the absorbed dose or administered activity maximizing P*, giving the most balanced benefit/risk ratio, provided that TCP and NTCP curves are available for the treatment. Figure 7 illustrates the relationship between the TI, TCP, NTCP, and P*.

## Discussion

The personalized dosimetry of ^90^Y-microspheres has been widely admitted to be the standard of care for treating tumours while preserving the liver parenchyma (1, 2, 20). While the partition model considers lung, liver and tumor as compartments, the other region-based and voxel-based methods allow for a further degree of personalization. In order to implemented these method, acquisition and reconstruction parameters needs to be optimized, otherwise this can lead to underestimation of absorbed doses to tumours (10). The choice of the dosimetry method should be done depending on the tools available and the training of the multidisciplinary team to personalized dosimetry. Several tools are available to understand how the irradiation conditions are related to a clinical outcome. There is a need to interpret clinical data from a mechanistic point-of-view, using logit or radiological models such TCP and NTCP to better optimize treatment planning. Tools such as EUD or NTCP have a great potential to tailor liver complication, but a clinical validation is still needed to extend their usage in clinical routine. Nevertheless they are interesting to understand the relationship between liver absorbed dose, the fraction of liver treated, microsphere activity load and potential liver failure thanks to their underlying biological concepts. The therapeutic index or the complication-free TCP are the state-of-the-art of optimizing treatment planning, to balance efficacy and toxicity in a quantitive way. However, they require accurate TCP and NTCP models that are still need to be developped for radioembolization. The availability of standardized clinical data should allow for the development these models.

## Conclusion

The treatment planning of selective internal radiation therapy with ^90^Y-microspheres can be tailored, thanks to well-established personalised dosimetry. Currently, at best, it relies on multicompartmental or voxel-based dosimetry. Considering parameters such as liver fraction, tumour histology, activity per sphere, liver underlying disease, and previous hepato-toxic therapies, along with radiobiological modelling, could potentially help to better guide treatment strategies.
