# Supplementary material for: Concepts and methods for the dosimetry of radioembolisation of the liver with Y-90-loaded microspheres
Source: Front Nucl Med. 2022 Sep 15;2:998793. doi: 10.3389/fnume.2022.998793 (PMC11464973; doi:10.3389/fnume.2022.998793)

Supplemental data

Table 1 : dataset of 100 subjects from 1 to 199 Gy (every 2 Gy) generated with response defined randomly with a binomial random variate generation, following a sigmoidal probability distribution given in equation

| **Subject #** | **Dose in Gy** | **Tumor response** | **Subject #** | **Dose in Gy** | **Tumor response** |
| --- | --- | --- | --- | --- | --- |
| 1 | 1 | FALSE | 51 | 101 | FALSE |
| 2 | 3 | FALSE | 52 | 103 | FALSE |
| 3 | 5 | FALSE | 53 | 105 | TRUE |
| 4 | 7 | FALSE | 54 | 107 | TRUE |
| 5 | 9 | FALSE | 55 | 109 | FALSE |
| 6 | 11 | FALSE | 56 | 111 | TRUE |
| 7 | 13 | FALSE | 57 | 113 | FALSE |
| 8 | 15 | FALSE | 58 | 115 | TRUE |
| 9 | 17 | FALSE | 59 | 117 | TRUE |
| 10 | 19 | FALSE | 60 | 119 | TRUE |
| 11 | 21 | FALSE | 61 | 121 | TRUE |
| 12 | 23 | FALSE | 62 | 123 | TRUE |
| 13 | 25 | FALSE | 63 | 125 | TRUE |
| 14 | 27 | FALSE | 64 | 127 | TRUE |
| 15 | 29 | FALSE | 65 | 129 | TRUE |
| 16 | 31 | FALSE | 66 | 131 | TRUE |
| 17 | 33 | FALSE | 67 | 133 | TRUE |
| 18 | 35 | FALSE | 68 | 135 | TRUE |
| 19 | 37 | TRUE | 69 | 137 | TRUE |
| 20 | 39 | FALSE | 70 | 139 | TRUE |
| 21 | 41 | FALSE | 71 | 141 | TRUE |
| 22 | 43 | TRUE | 72 | 143 | FALSE |
| 23 | 45 | FALSE | 73 | 145 | TRUE |
| 24 | 47 | FALSE | 74 | 147 | TRUE |
| 25 | 49 | FALSE | 75 | 149 | TRUE |
| 26 | 51 | FALSE | 76 | 151 | TRUE |
| 27 | 53 | FALSE | 77 | 153 | TRUE |
| 28 | 55 | TRUE | 78 | 155 | TRUE |
| 29 | 57 | FALSE | 79 | 157 | TRUE |
| 30 | 59 | TRUE | 80 | 159 | TRUE |
| 31 | 61 | TRUE | 81 | 161 | TRUE |
| 32 | 63 | TRUE | 82 | 163 | TRUE |
| 33 | 65 | FALSE | 83 | 165 | TRUE |
| 34 | 67 | TRUE | 84 | 167 | TRUE |
| 35 | 69 | FALSE | 85 | 169 | TRUE |
| 36 | 71 | FALSE | 86 | 171 | TRUE |
| 37 | 73 | FALSE | 87 | 173 | TRUE |
| 38 | 75 | TRUE | 88 | 175 | TRUE |
| 39 | 77 | TRUE | 89 | 177 | TRUE |
| 40 | 79 | FALSE | 90 | 179 | TRUE |
| 41 | 81 | TRUE | 91 | 181 | TRUE |
| 42 | 83 | FALSE | 92 | 183 | TRUE |
| 43 | 85 | TRUE | 93 | 185 | TRUE |
| 44 | 87 | TRUE | 94 | 187 | TRUE |
| 45 | 89 | TRUE | 95 | 189 | TRUE |
| 46 | 91 | TRUE | 96 | 191 | TRUE |
| 47 | 93 | FALSE | 97 | 193 | TRUE |
| 48 | 95 | TRUE | 98 | 195 | TRUE |
| 49 | 97 | TRUE | 99 | 197 | TRUE |
| 50 | 99 | TRUE | 100 | 199 | TRUE |

Figure 1 : Graphical representation of the reponders and non-responders according tumor absorbed dose. Responders value =1, non responders = 0.


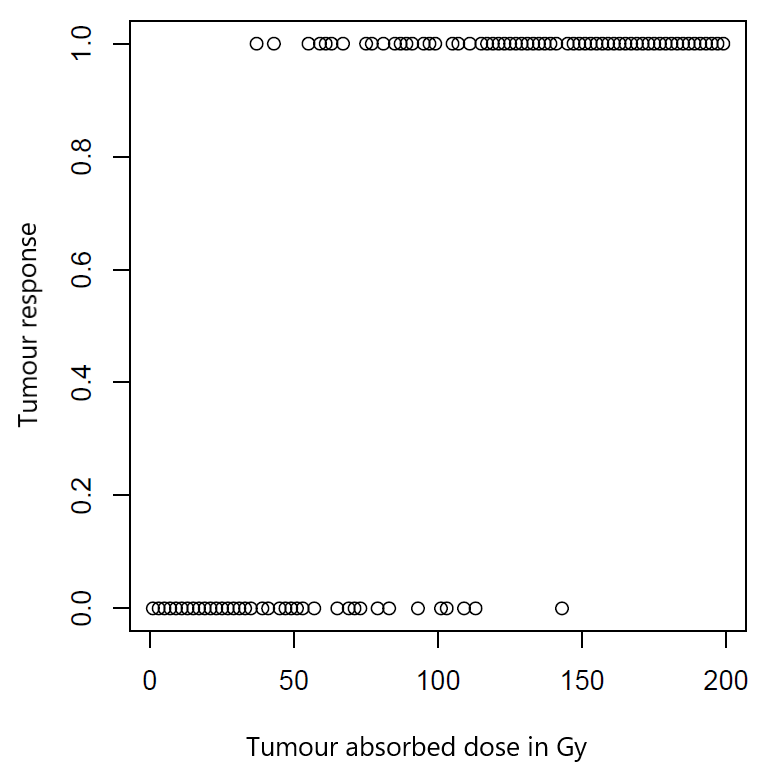

Supplement: Supplementary file 2 [file Datasheet2.docx]
