# Supplementary figures and images for: Concepts and methods for the dosimetry of radioembolisation of the liver with Y-90-loaded microspheres
Source: Front Nucl Med. 2022 Sep 15;2:998793. doi: 10.3389/fnume.2022.998793 (PMC11464973; doi:10.3389/fnume.2022.998793)

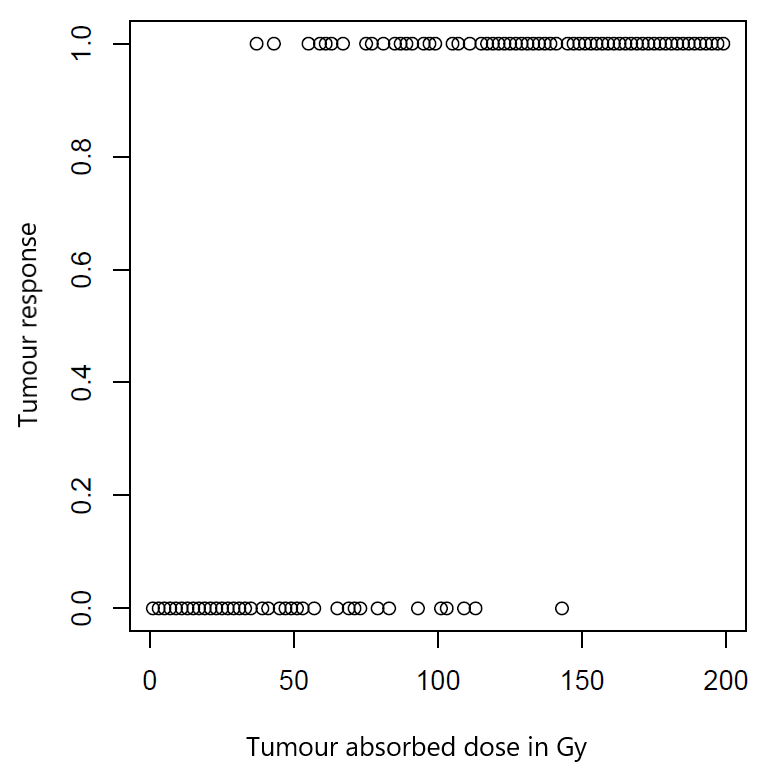

Supplement: Supplementary file 3 [file Image1.tiff]
